# Expression of microRNAs in the detection and therapeutic roles of viral infections: Mechanisms and applications

**DOI:** 10.1016/j.jve.2025.100586

**Published:** 2025-03-12

**Authors:** Mohsen Poudineh, Omeed Darweesh, Mohsen Mokhtari, Omid Zolfaghari, Azad Khaledi, Ahmad Piroozmand

**Affiliations:** a Infectious Diseases Research Center, Kashan University of Medical Sciences, Kashan, Iran; b Department of Microbiology and Immunology, Faculty of Medicine, Kashan University of Medical Sciences, Kashan, Iran; c College of Pharmacy, Al-Kitab University, Kirkuk, 36015, Iraq; d Laboratory Department, Paramedical School, Kashan University of Medical Sciences, Kashan, Iran; e Autoimmune Diseases Research Center, School of Medicine, Kashan University of Medical Sciences, Kashan, Iran

**Keywords:** MicroRNA, Epstein, Barr virus, Hepatitis C, Hepatitis B, Human immunodeficiency virus

## Abstract

In recent years, microRNAs (miRNAs) are potential diagnostic and therapeutic agents for viral infections. Here, we aimed to investigate the expression of microRNAs in the identification and treatment of viral infections.

MiRNAs are non-coding molecules that control gene expression and participate in numerous biological processes, including host immunity and pathogen duplication. MiRNAs have played a role in the pathogenesis of various viral infections, such as HIV and HCV. Their presence in the tissues and serum of infected patients has been demonstrated to help predict disease progression, identify disease subtypes, and evaluate treatment responses.

Research has shown that miRNAs can detect viral infections by identifying specific miRNAs in serum. For example, miRNA expression profiling was recently used to distinguish between hepatitis C and hepatitis B viral infections precisely. Furthermore, miRNAs can be used to detect the presence of multiple viral infections simultaneously by assessing the expression levels of these miRNAs. Also, miRNAs can differentiate between different genetic variants of the same virus, which is useful for identifying emerging viral strains or drug-resistant ones.

MiRNAs have been identified as being a factor in treating viral infections. For example, miRNA mimics have decreased gene expression and halted viral replication in HIV, HCV, and EBV. Moreover, microRNA antagonists have been utilized to inhibit pro-inflammatory cytokines, thereby modulating the immune response and the severity of infections.

## 1 Introduction

MicroRNAs are non-coding RNA molecules that function as critical regulators in the modulation of gene expression. They have acquired attention in recent years for their potential use in the finding of viral diseases. Recent studies recommend that miRNAs can be used to observe the presence of certain viruses, as well as to evaluate the severity of viral infections. This is due to their ability to designate the levels of expression of certain genes associated with a specific virus. Moreover, miRNAs can also be used to differentiate between active and latent infections, helping doctors make more accurate diagnoses.^1^^,^^2^

Human infectious diseases such as HIV, tuberculosis, malaria, and influenza are responsible for a tremendous burden of morbidity and mortality globally. With the advances in medicinal science and technology, understanding the biology of these infectious diseases through biomarkers has become essential for diagnosis. Biomarkers have the potential to provide valuable information about an individual's susceptibility to or progression of human infectious diseases. Furthermore, potential and emerging biomarkers may predict or assess the presence of a disease before symptoms arise or alert us to its presence, we could even monitor the response to treatment and so on. This development has no doubt added a new dimension to our understanding of human infectious diseases, one that can potentially save lives in this age where global pandemics are on the rise.^3^

MicroRNAs also play a significant role in biomarker research for liver diseases.^4^ They are small, non-structural molecules that have the potential to identify changes in gene expression before and during disease development. They have been extensively studied and found to be effective indicators of various types of liver diseases such as Hepatic Cirrhosis, hepatocellular carcinoma, and fatty liver disease. MicroRNAs exhibit the capacity to identify nuanced modifications in market signaling pathways that cannot be detected by conventional methods. This makes them powerful tools for predicting the early onset of liver disease before clinical symptoms appear, allowing medical professionals to better monitor, diagnose, and treat patients more efficiently and effectively.^5^

The diagnostic perspective in medicine is rapidly changing with advances in technology. With the help of new techniques and tools, medical practitioners can diagnose and treat diseases more accurately and efficiently. This has opened up a whole new frontier for medical research, allowing for the development of better treatments and cures for various illnesses. As medical practitioners continue to explore this field, they are discovering new ways to diagnose diseases faster, more accurately, and with fewer side effects. This is revolutionizing the way we look at medicine today and paving the way for a brighter future in healthcare.

The immune system is a natural defense mechanism of the body against pathogenic invasions and disease. Knowing the potential markers of infection and immune status can help us better understand how to protect ourselves from illnesses.^6^ By understanding these markers, we can take proactive steps to improve our health and reduce the risk of infection.

In this review, we study the types of microRNAs involved in viral diseases and their potential uses in the therapeutic administration and detection of viral infections, specifically those caused by HBV, HCV, HIV, EBV, RSV, and Coxsackie virus.

### 1.1 The role of the microRNA in HBV and HCV infections

The main causes of viral hepatitis are hepatitis B and hepatitis C viruses, which are a significant risk to the public's health. Hepatocellular carcinoma (HCC) may develop as a result of oncogenic alterations that result from chronic infection-induced liver inflammation and damage. Currently, there is a growing body of evidence that suggests that aberrant miRNA expression is a significant factor in the pathophysiology of viral hepatitis.^7^

### 1.2 MicroRNA signaling in HBV infection

**Fig. 1:**
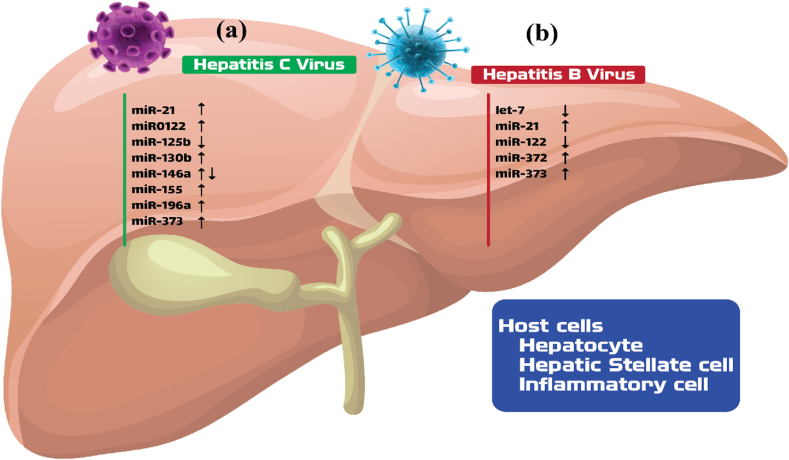
Types of microRNAs involved in hepatitis B and hepatitis C virus infections; In Figure **(a)**, microRNAs whose expression decreases under the influence of hepatitis C (including miR-125, miR-146a) and microRNAs whose expression increases in liver cancer tissue (miR-21, miR- 122, miR-130b, miR-146a, miR-155, miR-196a, miR-373) and in Figure **(b)**, the microRNAs whose expression changes under the influence of hepatitis virus have been identified, which are in the order of microRNA mir-372, miR-373 and miR-21 expression increases and let-7 and miR-122 expression decreases. These changes help in timely diagnosis of infection caused by these viruses; For example, miR-21 and miR-122 expression changes play an essential role in the early diagnosis of liver cirrhosis.^46^

Acute hepatitis caused by the Hepadnaviridae family of DNA viruses, including the hepatitis B virus, can progress to chronic illness in roughly 5 % of cases.^8^ The persistent inflammatory state and tumorigenic signaling that HBV induces can lead to hepatocellular carcinoma (HCC).^9^ As abstracted in Table 1, although the purpose of miRNAs related to HBV is unknown, Numerous investigations have shown that decreased cellular miRNA expression is significant during HBV infection and oncogenesis.^7^ A liver-specific miRNA called MiR-122 plays a key role in tumor suppression, the ongoing preservation of a healthy liver, and the metabolism of cholesterol (Fig. 1). MicroRNA-122 has been found to have a negative effect on HBV replication, resulting in increased p53 activity by downregulating CCNG1 (Cyclin G1). It has been found that miR-122 has a significant anticancer impact by controlling the Wnt/β-catenin signaling.^10^ Moreover, It has been suggested that miR-122 may impede the epithelial-mesenchymal transition (EMT) and hepatocellular carcinoma cell movement by damaging the RhoA/Rock pathway, which is vital for cytoskeletal events such as fiber bundles generation.^11^ The researchers employed RhoA and Rac1 as luciferase reporter plasmids that had miR-122 binding sites. The findings revealed that miR-122 had a significant effect on the total and active levels of Rac1 and RhoA.^12^ Furthermore, HBV replication can be suppressed by regulating the IFN signaling pathway.^13^

**Table 1:** Characteristics of microRNA obtained from selected studies.

miRNA	Source	Characteristics	Pathways targeted	References
miR-155	Host	Stimulator of Type I Interferon signaling pathway Increases the innate and adaptive immune response Antiviral functions Inhibitor of the HBV replication Higher levels in HIV infected cells Inhibition of NF-κB signaling pathway – promotion of viral latency Higher levels in HCV infection → increase the activity of	Type I Interferon signaling pathway NF-κB signaling pathway Wnt/β-catenin signaling	[^14^,^15^]
miR-122	Host	Stimulator of Type I Interferon signaling pathway Increases the innate and adaptive immune response Antiviral functions – Liver-specific Antiviral functions in HBV infection – Inhibition of HBV replication Diminished levels — promotion of viral persistence and oncogenesis Antitumorigenic effects by regulation of Wnt/β-catenin pathway Impairment of RhoA/Rock pathway	Type I Interferon signaling pathway Wnt/β-catenin pathway RhoA/Rock pathway	[^16^,^17^,^18^]
miR-146	Host	Increased in HIV and HCV infections Decreased activity of the NF-κB signaling pathway Proviral functions	NF-κB signaling pathway	^19^
miR-21	Host	Increased in HIV and HCV infections Decreased activity of the NF-κB signaling pathway Proviral functions in HIV and HCV infection Antiviral functions in Coxsackievirus B3 and SARS-CoV-2 infection Decreasing its level in HPV infections → downregulation of STAT3	NF-κB signaling pathway MAP2K3/p38 MAPK pathway STAT3 signaling pathway	[^20^,^21^]
hsa-miR-483–3p	Host	Up-regulated by HCV Increase the activity of PI3K/Akt signaling pathway, prolonging cell survival Proviral functions	PI3K/Akt signaling pathway	[^22^,^23^]
hsa-miR-320c	Host	Up-regulated by HCV Increase the activity of PI3K/Akt signaling pathway, prolonging cell survival Proviral functions	PI3K/Akt signaling pathway	[^22^,^24^]
miR-199a-5p	Host	Proviral functions Low levels lead to a decrease of viral replication in HCV infection Low levels lead to a decrease of viral replication in HCV infection	PI3K/Akt signaling pathway	[^25^,^26^]
miR-125b	Host	Suppressor of PI3K/Akt signaling pathway	PI3K/Akt signaling pathway	[^27^,^28^]
miR-499a	Host	Proviral functions in HCV infection	Notch signaling pathway	^29^
miR-BART7-3p	Viral - produced by EBV	Regulatory functions for the PI3K/Akt/GSK-3β pathway Aberant regulation of Wnt pathway → excessive cellular proliferation	Wnt signaling pathway	[^30^,^31^]
miR-BART1	Viral -produced by EBV	Activation of PI3K/Akt/GSK-3β pathway	PI3K/Akt/GSK-3β pathway	[^30^,^32^]
miR-BART16	Viral -produced by EBV	Inhibition of IFN signaling pathway Proviral function → increased replication	IFN signaling pathway	[^33^,^34^]
miR-BART19–3p, miR-BART17–5p, miR-BART14, miR-BART18–5p	Viral -produced by EBV	Inhibition of Wnt pathway inhibitory genes	Wnt signaling pathway	[35, 36, 37]
miR-BART22	Viral -produced by EBV	Viral oncogenesis in NPC and immune evasion Immune surveillance escape; T-cell differentiation, activation and recognition	LAMP2A NDRG1; IL12	[^38^,^39^]
miR-132	Host	Downregulated in HBV infection → enhanced carcinogenic feature of HBV Normal levels → inhibition of HCC cell proliferation	Akt-signaling pathway	[^40^,^41^,^42^]
miR-372 miR-373	Host	Upregulated in HBV infection Levels correlated with the number of HBV DNA copies Proviral functions → increased viral expression and replication during HBV infection	NFIB-dependent pathway	[^40^,^43^, ^44^, ^45^]

It has been demonstrated that miR-132 has tumor-suppressive attributes in a variety of cancer types.^40^ However, The ability of HBV to cause cancer is enhanced by the decrease in expression of the HBx protein during HBV infection.^47^ Scientists have shown that microRNA132 disrupts the PI3K-Akt signaling pathway,^48^ significantly reduced the proliferation of HCC cells. Also, it's discovered that the expression of Phosphorylated Akt, cyclin D1, Phospho-GSK-3β, and β-Catenin was significantly reduced in carcinogenic cells after the application of miR-132,^49^ the results indicate associating the Akt signaling pathway and microRNA 132 in the development of liver cancer (Table 1).

In a variety of human cancers, microRNAs 371 and 373 may not function as anti-oncogenes. The elevation of miRs-372–373 in HBV infection correlates favorably with the amount of HBV genome copies. MicroRNAs 372 and 373 can also improve HBV production and replication by targeting an NFIB-related pathway, which can boost the spread of the virus^7^ (Fig. 1).

### 1.3 MicroRNA signaling in HCV infection

Similar to HBV, the hepatitis C virus (HCV) infects people and causes chronic viral hepatitis in 60–80 % of cases.^50^ Approximately 20 % of individuals with chronic hepatitis C are susceptible to developing hepatocellular carcinoma. Due to the error-prone manner in which the HCV RNA-dependent RNA polymerase (RdRp) functions, a large number of viral quasi-species exist. The consequent heterogeneity of HCV poses significant challenges to the development of new medications and potent vaccines as well as to diagnosis.^51^

MicroRNAs affected by hepatitis B are primarily involved in DNA repair, transcription, and apoptosis, while those suppressed by HCV are primarily involved in immune modulation, antigen presentation, and cellular division cycle. Scientists have suggested that some cellular miRNAs might be reduced during the defense against RNA viruses. It has been observed that HCV infection has a higher concentration of down-regulated miRNAs than HBV infection (Fig. 1), for example, some studies have demonstrated that Wnt1, which has two miR-152 binding sites on the 3′-untranslated region (3′UTR) of Wnt signaling pathways, functions as a targeted gene. It has been concluded that miR-152 expression is downregulated significantly due to the overexpression of the HCV core protein. This down-regulation usually inhibits Wnt1 in HCV infection.^20^ The Wnt/β-catenin pathway then becomes more active, which promotes excessive cell division. The scientists have suggested that overexpressed miR-155, which is found in Hepatitis C infection, can enhance Wnt/β-catenin signaling (Table 1). The multimeric structure of the Wnt signaling pathway comprises the Glycogen synthase kinase-3 beta enzyme, the Axin protein, and the adenomatous polyposis coli. The phosphorylation of Beta-catenin by this structure is what causes it to be degraded by the ubiquitin-proteasome pathway. The Wnt/Wnt/β-catenin signaling pathway was activated by miR-155, which resulted in a significant reduction in APC levels.^19^^,^^52^

However, HCV infection also causes several miRNAs to be upregulated. MiR-21 is one of these miRNAs that is increased by HCV. To evade the immune response, MiR-21 targets components of the TLR signaling pathway, such as interleukin-1 receptor-associated kinases 1 and 2, MyD88, and TRAF6.^19^ MiR-122 can also be up-regulated during HCV infection, which indicates that IFN therapy will not prove to be as effective. MiR-122 overexpression can result in an increase in SOCS3 expression and a decrease in STAT3 activation, which in turn results in a decrease in antiviral gene stimulation through interferon-stimulated gene factor 3. MiR-373, which inhibits the JAK/STAT signaling cascade by targeting IRF9 and JAK1, further promotes IFN resistance. By boosting T cells, they are involved in the inflammatory response during HCV infection. MiR-155 increases autoimmune inflammation and is implicated in the activation of multiple inflammatory mediators.^12^^,^^53^ MiR-155 expression is induced by HCV both in vitro and in vivo. In serum samples and liver tissue, HCV-infected individuals exhibit a greater amount of miR-155 compared to uninfected controls.^16^ The expression of miR-155 considerably decreases in patients who successfully eliminate HCV after treatment, endorsing the idea that miR-155 is upregulated to facilitate HCV infection.^14^ MiR-155 regulates the immune response during chronic HCV infection by modulating Natural Killer Cell activity and affecting the expression of interferon-regulated genes, thus influencing the immunological response to Hepatitis C.^17^

Hepatitis C infection leads to an increase in the expression of MicroRNA-21. This microRNA plays a crucial role in controlling the targeting components of the TLR signaling pathway, including proteins such as IRAK4, TRAF6, IRAK1, and MyD88 in liver tissues. As a result, the immune response is prevented and the production of Interferon type I is suppressed^54^(Table 1).

**Fig. 2:**
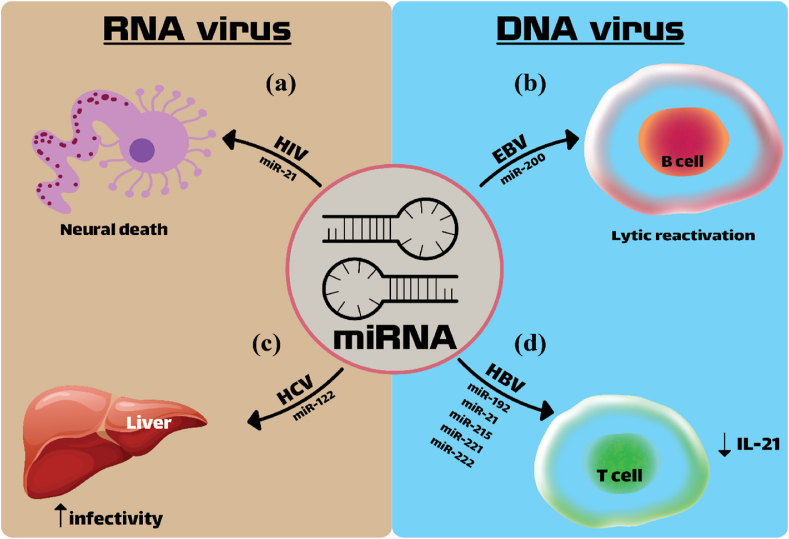
MicroRNA expression changes in viral infections and their consequences; HIV infection increases the expression of miR-21, which activates the TLR7-dependent death pathway, which causes neurotoxicity **(a)**.^56^ In infections caused by EBV, the expression of microRNAs of the miR-200 family, which play a role in inhibiting tumor progression and metastasis, is reduced. It is also known that members of miR-200 are involved in lytic reactivation in epithelial cells and lymphocytes. Infected B cells have a role, so that the reduction of the expression of this miRNA reduces the lytic reactivation **(b)**.^57^ MicroRNA 122 is a specific liver microRNA that has the role of a tumor suppressor, which increases the infectivity of this virus by helping to stabilize the genome and accumulate the viral genome **(c)**.^58^ In hepatitis B infection, an increase in miR-221, miR-222, miR-215, miR-21, miR-192 is observed, which causes a decrease in interleukin 21 produced by T lymphocytes **(d)**.^59^

MiR-122, a microRNA that is specific to the liver, is a marker of poor response to IFN therapy and can be elevated by HCV infection. Increased expression of it tends to reduce STAT3 activation, increase SOCS3 expression, and promote antiviral-related genes via ISGF3^55^ (Fig. 2).

HCV infection may have a complex relationship with the expression levels of MicroRNA-146a. Peripheral blood mononuclear cells (PBMCs) are naturally abundant in MiR-146a, which regulates immunologic response. It was previously shown that the expression of MiR-146 increases in hepatocytes that are infected with hepatitis C. It was also discovered that MiR-146a expression was reduced in PBMCs from chronic HCV patients. This is partially clarified by variations in genotypes of hepatitis C.^60^

It appears that MiR-130a plays a dual role in HCV infection. Compared to healthy individuals, people with HCV infection have substantially higher levels of its expression in liver tissues. The inhibition of miR-130 expression causes an increase in IFITM1, which then inhibits the replication of Hepatitis C in hepatocytes.^61^ The innate immune response that results from downregulating IFITM1 and upregulating miR-130a can be used by HCV to maintain chronic infection.^62^ Hepatitis C replication is inhibited in both the JFH1-based cell culture system and the Con1b replicon when miR130a is overexpressed.^63^ MiR-130a is responsible for reducing miR-122 and elevating the expression of proteins that control immune responses, including Interferon type I, USP18, ISG15, and MxA. According to these findings, miR-130a plays a role in HCV replication and the host's immune response.^3^^,^^64^

The inhibition of HCV gene expression and replication is achieved by inducing hemoglobin oxygenase 1 expression and inhibiting Bach1 expression with MicroR-196a.^65^ In patients with chronic hepatitis C, miR-196a expression levels are significantly lower than those in patients who don't have HCV or alanine aminotransferase, which suggests that miR-196a release decreases in hepatocytes infected with HCV. As a result, microRNA-196a has the potential to predict chronic HCV infection in its early stages. Furthermore, hepatoma cells can increase their expression of microRNA-196a after exposure to IFNβ.^66^^,^^67^ MiR-373 also decreases the JAK/STAT pathway, which leads to an increase in IFN resistance.^68^

### 1.4 The role of the MicroRNA in respiratory syncytial virus infection

The respiratory syncytial virus (RSV) is a viral infection that is commonly found in youngsters and older adults, causing an important number of hospitalizations, medical visits, and an annual global casualty rate exceeding 14,000. This virus harbors a negative-sense single-stranded RNA (ss RNA) genome, which spans a length of 15 kilobases (kb) and codes for a total of 11 proteins. These proteins, namely NS1, NS2, M, N, P, L, F, G, SH, M2-1, and M2-2, are pivotal in the viral life cycle and pathogenesis. Despite extensive efforts by the scientific community over the past six decades, the development of a viable RSV vaccine has proven to be an elusive goal, with existing prevention and treatment methods falling short of effectiveness. The primary hindrance to progress in this field stems from the limited understanding of the intricate interactions between the RSV and its host, thus impeding the success of various experimental tests and investigations.^69^

RSV infection has been observed to have a significant impact on the expression of miR-221 in the HEp2 cell line as well as in HBEC culture. This finding suggests that RSV infection leads to a decrease in the expression levels of miR-221 in these specific cellular environments. Furthermore, a separate study has discovered that RSV can upregulate the NGF-TrKA axis in the human airways. This upregulation is achieved through the inhibition of miR-221 expression. This inhibition, in turn, facilitates the promotion of viral multiplication by interfering with the normal apoptotic processes of infected cells. Therefore, RSV infection can disrupt the natural cellular defense mechanism of apoptosis by specifically targeting the expression of miR-221.^70^

RSV induces the expression of miR in at least two mechanisms, as elucidated by previous scientific investigations. In the first mechanism, both let-7b and let-7i are activated in response to IFN-β in human monocyte-derived dendritic cells (MDDCs) and HBECs, respectively. Furthermore, the second mechanism involves the induction of miR-30b in HBECs, independent of IFN, but through the involvement of NF-κB. Notably, following 48 h of RSV infection of HBECs in an in vitro setting, the expression of miR-30b and let-7i exhibits a significant increase. It is important to highlight that this increase occurs specifically in HBECs infected with RSV, excluding the presence of NS1 and also NS2 proteins (44), the normal HBEC line cells exhibit an increased expression of miR-30b and let-7i. The NS1 and NS2 proteins may disrupt the up-regulation of let-7i and miR30b expression by inhibiting the production of type I IFNs and other cytokines involved in miRNA transcription. Moreover, miR let-7 plays a vital role in inducing host genes during viral infection.^71^ Another investigation revealed indications that the RSV G protein facilitates the generation of let-7f, thereby impeding CCND1 and DYRK2 and fostering viral replication by inducing cellular arrest in G1, consequently halting the cell cycle.^72^

MiR-453, miR-339–5p, miR-27a, and miR-574, which are all excessively expressed following respiratory syncytial virus (RSV) infection, have been identified as part of the group of microRNAs that exhibit dysregulation in response to this viral pathogen.

IFNAR1 expression can be directly downregulated by miR-29a by targeting the IFNAR1 3′ UTR. Additionally, RSV NS1 inhibits IFNAR1 expression in lung adenocarcinoma cell line A549 at both the protein and RNA amounts. The findings imply that miR-29a increased during infection with RSV, adversely controls IFNAR1, and is essential for the replication of the virus caused by RSV NS1. RSV replication is aided by RSV NS1's interaction with KLF6 and modulation of the levels of miR-24 and TGF- β. Eight mimics-miR-744, miR-542–5p, miR-124a, miR-346, miR-155, miR-128a, miR-452, and miR-28-show a >75 % decrease in both of the RSV strains.^73^ One of them, miR-155, has been shown to control innate immunity by promoting the expression of genes that IFN induces.^74^ Also during RSV infections, miR-155 expression is higher in peripheral blood than healthy controls. This suggests that miR-155 may be involved in the body's response to RSV infection^75^ (Table 1).

### 1.5 The role of the MicroRNA in covid 19 infection

**Fig. 3:**
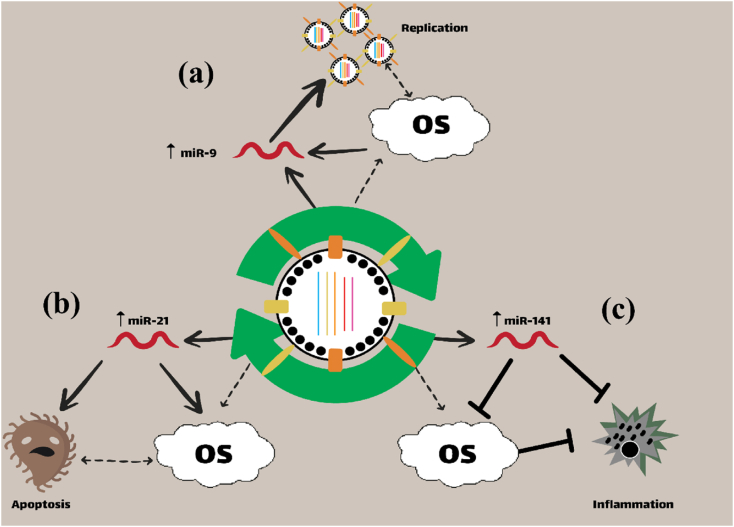
MicroRNAs involved in coxsackie virus infection, miR-9, which has an essential role in the formation and maturation of neural progenitor cells, and may play a role in inhibiting the growth of coxsackie and influenza viruses **(a)**.^91^^,^^92^ MiR-21 increase by virus-infected cells inhibits the growth of coxsackie virus B3 and SARS-CoV-2 through the MAPK pathway and also inhibits the apoptosis of the involved cells **(b)**.^7^ MicroRNA 141 plays an important role in suppressing metastasis and growth of HCC cells, and infection with Coxsackie B3 and influenza viruses increases the expression of miR-141, which accelerates inflammation and leads to oxidative stress **(c)**.^93^

COVID-19, attributable to the SARS-CoV-2 virus, represents a highly contagious respiratory illness that emerged in late 2019 and subsequently led to a global pandemic. This pandemic has profoundly impacted health systems, economies, and daily life on a worldwide scale.^76^ The involvement of microRNAs (miRNAs) in the infection process, host immune response, viral replication, and disease severity of covid 19 is noteworthy.^77^ MiRNAs play a crucial role in regulating immune responses to pathogens by modulating the expression of genes associated with immune signaling pathways.^78^ For instance, miR-146a is essential for regulating immune functions and inflammation; elevated levels of miR-146a can modify cytokine production.^77^^,^^79^ In vitro studies by Li et al.^80^ and in vivo studies by Boldin et al.^81^ highlight the critical role of miR-146a in managing immune responses and inflammation. Acting as a molecular brake, miR-146a controls excessive immune activation, which is essential to prevent harmful inflammatory responses. By targeting key adapter proteins in inflammatory pathways, miR-146a helps maintain immune balance and prevents the development of inflammatory and autoimmune diseases. Additionally, miR-21 has been implicated in the regulation of immune responses, research indicates an increasing level of miR-21 in severe cases, suggesting its involvement in driving inflammatory responses^82^ (Fig. 3). MiR-155, recognized for its role in enhancing the immune response, may contribute to the hyper-inflammatory reactions observed in severe COVID-19 cases, which can lead to a ‘cytokine storm.'^83^ (Table 1). A study by Alessandra Giannella et al.^84^ explored circulating microRNA signatures and found that a combination of high serum levels of miR-21–5p and miR-22–3p, along with low levels of miR-155–5p and miR-224–5p, could distinguish severe COVID-19 cases from mild/moderate ones. This suggests that these microRNAs together can serve as potential biomarkers for disease severity and outcomes in COVID-19 patients Certain miRNAs possess the capacity to bind to the SARS-CoV-2 genome, potentially inhibiting viral replication.^85^ For example, miR-34a regulates apoptosis and influences immune responses; variations in its expression may affect the body's ability to combat the virus.^86^ Alterations in miRNA expression are associated with the severity of COVID-19, with specific miRNAs frequently upregulated in severe cases. Dysregulation of miR-200c may contribute to the pathogenesis of COVID-19 b y influencing immune reactions and inflammatory pathways.^87^ Given their significant roles in immune responses and disease progression, miRNAs have emerged as promising biomarkers for the early detection of COVID-19. Distinct miRNA signatures can accurately differentiate COVID-19 from other respiratory infections.^88^^,^^89^ Ongoing research endeavors aim to further illuminate the involvement of miRNAs in COVID-19, potentially leading to innovative diagnostic and therapeutic strategies for enhanced patient management during this global pandemic.^90^

### 1.6 The role of the MicroRNA in influenza infection

Influenza, commonly referred to as the flu, is a contagious respiratory illness caused by influenza viruses. There exist four types of influenza viruses: A, B, C, and D. Among these, Influenza A and B viruses are primarily responsible for seasonal flu epidemics.^94^ Influenza A viruses can be further classified into subtypes based on two surface proteins: hemagglutinin (H) and neuraminidase (N).^95^ Historical notable influenza pandemics, including the Spanish flu of 1918, the Asian flu of 1957, and the H1N1 pandemic of 2009, were attributed to various subtypes of Influenza A viruses.^96^ Recent research has indicated that microRNAs (miRNAs) play a significant role in influencing both the host's immune response and the replication dynamics of the virus. Studies have demonstrated that miRNAs can modulate the host's antiviral response by targeting specific genes implicated in the immune system. For instance, certain miRNAs may suppress the expression of critical genes necessary for an effective antiviral response, thereby facilitating viral replication. Conversely, other miRNAs have been found to enhance the host's defense mechanisms by upregulating the expression of antiviral genes.^97^ Moreover, the influenza virus can encode viral miRNAs that interact with host cellular machinery to promote viral replication and evade immune detection. These viral miRNAs interfere with the host's gene expression, thereby creating a conducive environment for viral proliferation. Specifically, miR-141 has been identified as a regulator of interferon (IFN) signaling and its associated genes, including MxA (myxovirus resistance protein A) and STAT3 (signal transducer and activator of transcription 3).^88^ miR-141 downregulates these genes, which are essential for the antiviral response, thus supporting the replication of the influenza virus and accelerating inflammation^88^^,^^98^ (Fig. 3). miR-323 has been shown to inhibit influenza A H1N1 replication by downregulating viral gene expression in infected cells. It contributes to the host's immune response by targeting genes involved in viral replication.^99^ Similarly, miR-491 also inhibits influenza A H1N1 replication through the downregulation of viral gene expression, thereby assisting in controlling the virus's replication within host cells.^97^ Additionally, miR-654 has been demonstrated to inhibit influenza A H1N1 replication by targeting viral genes and modulating the host's immune response, playing a role in the regulation of the host's antiviral defense mechanisms.^97^ Understanding the role of miRNAs in influenza virus infection presents new opportunities for therapeutic interventions. Targeting specific miRNAs with inhibitors or mimics may effectively modulate the host's immune response and limit viral replication, thereby offering a novel approach to antiviral therapy.^100^

### 1.7 The role of the MicroRNA in HIV-1 infection

HIV-1 primarily infects CD4^+^ T cells by recognizing the CD4^+^ receptor on the surface of the cell. The virus steadily decreases the amount of CD4^+^ T cells in patients, impairing the immune system and finally leading to acquired immunodeficiency syndrome (AIDS). Various host factors, including miRNAs, regulate HIV-1 replication inside host cells. MiRNAs may directly target virus mRNAs or influence the production of host proteins that HIV-1 steals for its replica.^101^

MiRNAs can detect HIV-1 infection and track disease advances. HIV-1 infection has been shown to alter the expression of numerous MicroRNAs, either through altering host cellular activity or as an effect of the cellular response to HIV-1 infection. The capacity of MicroRNA analyses to discriminate between people with and without HIV-1 infection has been observed all around the world.^102^

MicroRNAs can influence HIV-1 infection through a variety of methods. Anti-HIV-1 miRNAs inhibit HIV-1 activity through the auxiliary receptors CCR5 or CXCR4 or target HIV-1 directly via the *gag*, *pol*, *env*, *vif*, and *tat* genes of the HIV-1 genome.^103^

Inside the HIV-1 genome, miR-324–5p, and miR-378 targeted the *env* gene, miR-149 targeted the *vpr*, and *vif* genes, miR-29b, and miR-29a targeted *nef* gene, miR-150, miR-125b, miR-28, miR-382–5p, and miR-223 targeted the 3′UTR region of the HIV-1 mRNA in cells, which reduces CD4^+^ T-cell _activation through the resting time and the targets of miR-1290 and miR-196b are the 3′UTR region of the HIV1 RNA genome. In the context of antiretroviral treatment (ART), suppression of these two miRNAs can activate latent HIV-1, clearing dormant viral reservoirs through virus-induced cytolysis and host antiviral immunological responses.^104^

miR-132 was shown to be substantially more abundant in activated CD4^+^ T cells than in steady-state CD4^+^ T cells. The overexpression of MiR-132 enhanced the replication of HIV-1 and rendered activated CD4^+^ T cells more prone to HIV-1 infection.^105^ MiR-34a and miR-217 may considerably enhance tat levels as well as bind to the SIRT1 gene's mRNA and decrease its production, hence amplifying the transcriptional activation arbitrated by *tat* gene.^105^^,^^106^

MicroRNAs exert their influence on the consequences of HIV-1 infection through an indirect mechanism. This is accomplished by selectively targeting HIV-dependent elements that interact with HIV-1, including the histone acetyltransferase P300 and the P300-CREB binding protein-associated factor (PCAF). These factors play a crucial role in the process of tat acetylation, which subsequently triggers the up-regulation of HIV-1 LTR transcription.^107^ PCAF is targeted by cell-associated miR-17/92, which suppresses viral replication.^107^ MiR198 hinders the replication of HIV-1 through the modulation of cyclin T1, an indispensable constituent of the eukaryotic RNA polymerase II elongation complex. The interaction of this protein with CDK9 (transcription elongation factor B, or p-TEFb) results in the formation of a heterodimer. The p-TEFb complexes promote viral transcription by interacting with tat and HIV-1 trans-activation-response (TAR) sites. Cyclin T1 exhibits a higher prevalence in cells infected with HIV-1, specifically in macrophages, as compared to mononuclear cells.^108^

### 1.8 Alterations in microRNA levels in peripheral blood mononuclear cells (PBMCs) and serum from patients are associated with HIV disease progression

In total, 63 distinct microRNAs showed alterations in levels in HIV-1-infected PBMCs, demonstrating that certain miRNAs are expressed differently in various groups (Table 1). Let-7e, miR-146b, miR-101, and miR-155, for example, were only noticed in the low CD4^+^ T-cell count and high-viral load groups. MiR-16, miR-146b, miR-150, miR-223, and miR191 levels in T cells lowered in PBMCs from patients with different disease progression characteristics by a ratio of three to nine. We contrasted long-term nonprogressors (LTNP), slow progressors, and quick progressors in terms of miRNA expression in PBMCs. When compared to plasma viral load, miR-382–5p levels in LTNP showed a favorable linear connection. It is possible to identify between LTNP and chronic progressors, as well as between non-infected and infected people, using a combination of miR-155–5p and miR-382–5p. miR-155–5p and miR-382–5p level decreases are advantageous for the prevention of illness progression.^20^

In the early stages of infection, 10–15 % of HIV-1-infected individuals have a quick decline in CD4^+^ T-cell count, which quickly progresses to AIDS. MiR-200c, miR-31, miR-99a, miR-526a, and miR-503 levels differed between rapid and slow progressors in the PBMCs, and these differences might be employed as biomarkers for fast progressors to support early interposition. MiR-19b, miR-615–3p, miR-146a, miR-382, miR-144, miR-34a, and miR-155, which regulate genes associated with innate immunity and inflammation, were noticeably upregulated in PBMCs from HIV-1-infected patients with high viral loads.^109^ To assess the levels of miR-146b-5p, miR-16, miR-191, miR-150, miR-146b-5p, and miR-223 expression in plasma in patients with AIDS, AIDS patients who had not received ART, ART recipients, and ART recipients who had proven drug resistance. In addition to identifying several progression groups, they discovered that microRNA-146b-5p levels in PBMCs and plasma also expected a response to ART. Additionally, microRNA-150 may be employed as a marker to track the development of the HIV/AIDS infection and the impact of ART. Among 165 individuals with chronic HIV-1 infection, there exists an inverse association between microRNA-29a (miR-29a) levels and both HIV viral load as well as the extent of immunosuppression (as indicated by the CD4^+^ T cell count and the CD4^+^ T/CD8+ T ratio). Patients differ in terms of their miR-29a levels and how they respond to therapy. Patients with low CD4 counts (CD4 350 cells/l) who failed to respond to therapy showed low levels of microRNA-29a. Thus, microRNA-29a can be employed as a biological marker of long-term survivors and ART patients, as well as to foretell the prognosis and development of illness.^109^

The disparity in microRNA-31 and miR-31 levels between PBMCs from patients with HIV-1 viremia and elite controllers mirrored the development of HIV-1 infection. MicroRNA-31 levels were shown to be favorably connected with CD4^+^ T-cell count, whereas microRNA-341–3p levels were found to be negatively associated with viral load. This finding shows that the amounts of these microRNAs might be used to predict the course of HIV-1 infection.^108^ To compare alterations in miRNA plasma levels in top controllers, those with tenacious HIV-1 infection, and healthy people. We discovered that plasma microRNA-33a-5p, microRNA-29b-3p, and microRNA-3146a-5p levels were considerably greater in top controllers than in chronic infection patients. In vitro research revealed that overexpression of miR-33a-5p and miR-29b-3p might suppress HIV-1 production. The elite controller and viremia groups showed differential expression of 23 distinct microRNAs. Among these microRNAs were miR-27a, miR-221, miR-29a, and miR-27a, in the serum of elite controllers and HIV-1-negative individuals, certain factors exhibited up-regulation. However, these same factors were down-regulated in patients with viremia and those receiving antiretroviral therapy (ART). Other microRNAs were also down-regulated in the serum of exclusive controllers and HIV-1-negative people and boosted in those of viremia-positive people and people on antiretroviral therapy (ART).^110^^,^^111^

In the context of HIV-1 infection, CD4^+^ T cells serve as the primary targets, the alteration of their miRNA profiles is much more directly associated with the development of the illness. During HIV-1 infection, the essential inflammatory cytokine interferon-inducible protein 10 (IP-10) can cause immunological dysfunction and disease development. By contrasting the human miRNA with the gene expressing IP-10 sequences. Six miRNAs had sequences that matched the IP-10 3′UTR. Additional investigation indicated that miR-21, one of these microRNAs, was decreased in monocytes during HIV-1 infection and was in charge of the rise in IP-10 levels that caused inflammation and the quick loss of CD4^+^ T cells, which is strongly associated with disease development^111^^,^^112^(Fig. 2).

### 1.9 The connection between microRNAs and disease development in HIV-1-infected individuals

The host miRNA has a vital role in HIV-1 latency. For example, the levels of microRNA-155 are altered in the CD4^+^ T cells of HIV-1-infected people in the LTNPs and untreated patient groups.^109^ The pleiotropic cytokine IL-10 is the target of the Let-7 family of miRNAs. Let-7 expression is decreased in chronic progressors. IL-2 also a key role in the maintenance of CD4^+^ T cell activation, survival, and replication. MicroRNA-9 levels in CD4^+^ T cells from people with persistent HIV-1 infection are lower than in healthy people or LTNPs. This drop in miR-9 levels causes a rise in mature protein 1 in B cells, inhibiting IL-2 synthesis and encouraging disease progression, indicating that miR-9 plays a role in HIV-1 latency maintenance. These discoveries imply that long-term surveillance of microR-155 might be highly useful for determining HIV-1 latency status in infected persons.^113^ MiRNA expression in cells and body fluids has been linked to the intensity and progression of HIV infection. HIV-1-infected individuals exhibit diverse disease progression profiles, including typical progressors, rapid progressors, slow progressors, long-term non-progressors (LTNP), and those in the early stages of antiretroviral treatment (ART). These classifications are based on CD4^+^ T-cell counts, viral load, and ART status. Some microRNAs are expressed differently in various groups, and their aberrant expression can act as a biomarker of disease development and give crucial prognostic clues.^113^^,^^114^ Progression in HIV/AIDS patients can be monitored using miR-150 as a potential biomarker. The levels of miR-150 were compared in PBMCs and plasma from healthy donors, symptomatic patients, asymptomatic patients, ART patients, and drug-resistant patients. They discovered that this specific miRNA was expressed differently in each of these groups.^115^

### 1.10 The role of the MicroRNA in EBV

The Epstein-Barr virus is the first tumor virus to be discovered in humans and can lead to various cancers. Epstein-Barr virus-associated gastric cancer (EBVaGC) can be classified as its subtype of gastric cancer according to molecular classification.^116^ EBV, which belongs to the herpesvirus family, has the potential to cause a range of tumors, such as lymphoma, nasopharyngeal carcinoma (NPC), and gastric cancer. (GC).^117^ EBV is believed to be the first virus to encode its miRNAs. There are two groups of EBV miRNAs: BHRF1 and BmHIA rightward transcripts (BARTs)^118^(Table 1). In EBV, there have been 25 precursor miRNAs and 44 mature miRNAs identified so far. Despite BART miRNAs accounting for 15 % of all miRNAs in EBVaGC cell lines, there are still many miRNA functions that are not clear.^119^ Among the microRNAs expressed by host cells, members of the miR-200 family as shown in Fig. 2 have been observed to play a role in the reactivation of the lytic cycle in epithelial cells and B lymphocytes infected with EBV, such that reducing the expression of this miRNA reduces lytic reactivation.^120^

The tumor suppressor gene RB1 was initially discovered in retinoblastoma. The cell cycle regulator RB plays a vital role in regulating the start of DNA replication and cell division. RB suppresses tumors by regulating the transcription of downstream genes of DNA replication enzymes via E2F transcription factors that coordinate G1/S cell cycle transition.^121^ The growth of tumors is facilitated by the absence of RB, which results in unregulated cell cycle progression. The genetic or functional inactivation of RB occurs in many human malignancies, such as retinoblastoma, prostate cancer, lung cancer, and breast cancer.^122^

The p21 gene, also known as WAF1/CIP1, is a crucial CDK inhibitor that helps regulate cell cycle progression and acts as a tumor suppressor. The cell cycle's progress is hindered by P21 when Cyclin-dependent kinase 4, CDK6, and CDK2/cyclin E are blocked. When p21 is overexpressed, cell development stops in the G2 phase.^123^

### 1.11 Relative expression of RB and p21 in EBV-negative gastric cancer (EBVnGC) and EBV-associated gastric cancer (EBVaGC) cell lines

Three EBVaGC cell lines (plate_number_1, GT38, and GT39) and four EBVnGC cell lines (HGC27, MKN45, plate_number_2, and AGS) were analyzed using Western blot to examine the protein expression of RB and p21 genes. According to the study's findings, RB and p21 protein expression were lower in the three EBV-associated gastric cancer (EBVaGC) cell lines as compared to the four EBV-negative gastric cancer (EBVnGC) cell lines. To eliminate the influence of genetic background, the researchers examined the expression of RB and p21 in both AGS cells and EBV-infected AGS cells. The results also revealed that the levels of RB and p21 protein in AGS-EBV cells were lower than in AGS cells.^124^

### 1.12 The role of the MicroRNA in Coxsackievirus infection

The p38 MAPK pathway is crucial in regulating CVB3-induced pathologies. P38 MAPKs are a group of MAPKs that are protein kinases and convert extracellular signals into intracellular responses. It has been proven by various studies that Coxsackievirus B3 activates p38 MAPKs, leading to inflammation, viral replication, and apoptosis in the cells affected. CVB3 cytotoxicity and life cycle in HeLa cells are impeded by the p38 MAPK inhibitor, which suggests that p38 MAPK signaling is crucial for CVB3 cytotoxicity and life cycle in the affected cells.^125^ In addition, the use of pharmacological inhibitors to stop p38 MAPK phosphorylation can effectively inhibit CVB3 replication and release in both vitro and vivo. Furthermore, MAP2K3 can directly activate p38 MAPK in the upstream.^126^ Therefore, A therapeutic approach for CVB3 infection may involve targeting the MAP2K3/p38 MAP pathway.^127^ In mice with myocarditis caused by Coxsackievirus, MicroRNA-21 is upregulated in their cardiac tissues and plays a role in cardiovascular development, and Malfunction is observed in various cardiovascular diseases, including fibrosis, cardiac hypertrophy, and myocardial infarction. MAP2K3 can be directly targeted by miRNA-21, and miRNA-21 imitation can have an effective impact on reducing its expression in HCC^128^ (Fig. 3). Therefore, it can be inferred that MicroRNA-21 was implicated in MAP2K3/p38 MAPK communication during CVB3 infection. As shown in Fig. 3, MicroRNA-9 and MicroRNA-141 also play a role in Coxsackievirus infections. MicroRNA-9 has a role in suppressing Coxsackievirus infection.,^93^ and MicroRNA-141 is increased in Coxsackievirus B3 infection, which leads to accelerated inflammation.^129^

The main limitations of this study are; we only included the articles that we found online in the aforementioned databases, we were not aware of unpublished studies, and that only articles that were published in English were enrolled in this review.

In recent years, there has been substantial progress in the application of microRNAs (miRNAs), offering opportunities for their use in research and clinical settings to identify and treat viral infections. MiRNAs play crucial roles in various biological processes, including viral infection and host defense. Both virus-related miRNAs and host miRNAs can modulate the interaction between viruses and host cells, affecting viral replication, latency, reactivation, pathogenesis, and the progression of viral diseases. Additionally, miRNAs can serve as biomarkers for the detection and prognosis of viral infections, reflecting viral load, immune status, and tissue damage in infected individuals.

MiRNAs can act as valuable biomarkers or mediators in the treatment of virus-related infections by inhibiting viral replication, enhancing host immunity, and reversing associated pathological changes. Their involvement in regulating gene expression during viral infections positions miRNAs as promising targets for innovative therapeutic strategies, potentially leading to more effective antiviral treatments and improved patient outcomes. However, several challenges and limitations remain in the use of miRNAs, including issues related to stability, endosomal escape, specificity, and adverse effects such as immune system activation. Extensive further research is essential to elucidate the mechanisms and roles of miRNAs in viral infections, their interactions with the immune system, and their potential applications in the diagnosis and treatment of viral infections.

## CRediT authorship contribution statement

**Mohsen Poudineh:** Formal analysis. **Omeed Darweesh:** Methodology. **Mohsen Mokhtari:** Investigation. **Omid Zolfaghari:** Software, Methodology. **Azad Khaledi:** Conceptualization. **Ahmad Piroozmand:** Writing – review & editing, Resources.

## Informed consent

Not applicable.

## Ethics committee approval

Not applicable.

## Funding

The authors declared that this study has received no financial support.

## Declaration of competing interest

The authors declare that they have no known competing financial interests or personal relationships that could have appeared to influence the work reported in this paper.

## Data Availability

Data will be made available on request.
